# Internet-Based Cognitive Behavioral Therapy and its Association With Self-efficacy, Depressive Symptoms, and Physical Activity: Secondary Analysis of a Randomized Controlled Trial in Patients With Cardiovascular Disease

**DOI:** 10.2196/29926

**Published:** 2022-06-03

**Authors:** Peter Johansson, Johan Lundgren, Gerhard Andersson, Erland Svensson, Ghassan Mourad

**Affiliations:** 1 Department of Health, Medicine and Caring Sciences Linköping University Norrköping Sweden; 2 Unit of Internal Medicine, Division of Diagnostics and Specialist Medicine Department of Health, Medicine and Caring Sciences Linköping Univerisity Norrköping Sweden; 3 Department of Behavioural Sciences and Learning Linköping University Linköping Sweden; 4 Department of Biomedical and Clinical Sciences Linköping University Linköping Sweden; 5 Swedish Defence Research Agency Linköping Sweden

**Keywords:** internet-based cognitive behavioral therapy, cardiovascular disease, depression, self-efficacy, physical activity, mental health, depression, digital health, online health, digital therapy, cognition, self-care, CVD, internet-based, cardiology, heart disease, cardiac health, cognitive behavioral therapy

## Abstract

**Background:**

In patients with cardiovascular disease (CVD), knowledge about the associations among changes in depressive symptoms, self-efficacy, and self-care activities has been requested. This is because such knowledge can be helpful in the design of behavioral interventions aimed to improve self-efficacy, reduce depressive symptoms, and improve performance of self-care activities in CVD patients.

**Objective:**

We aim to evaluate if internet-based cognitive behavioral therapy (iCBT) improves self-efficacy and explore the relationships among changes in depressive symptoms, self-efficacy, and physical activity, as well as the influence of iCBT on these relationships.

**Methods:**

This study received funding in January 2015. Participant recruitment took place between January 2017 and February 2018, and the main findings were published in 2019. This study is a secondary analysis of data collected in a randomized controlled study evaluating the effects of a 9-week iCBT program compared to an online discussion forum (ODF) on depressive symptoms in patients with CVD (N=144). Data were collected at baseline and at the 9-week follow-up. Analysis of covariance was used to evaluate the differences in self-efficacy between the iCBT and ODF groups. Structural equation modeling explored the relationships among changes in depressive symptoms, self-efficacy, and physical activity, as well as the influence of iCBT on these relationships.

**Results:**

At follow-up, a significant difference in the increase in self-efficacy favoring iCBT was found (*P*=.04, Cohen *d*=0.27). We found an indirect association between changes in depressive symptoms and physical activity (*β=*–.24, *P*<.01), with the change in self-efficacy acting as a mediator. iCBT had a direct effect on the changes in depressive symptoms, which in turn influenced the changes in self-efficacy (*β*=.23, *P*<.001) and physical activity (*β*=.12, *P*<.001).

**Conclusions:**

Self-efficacy was improved by iCBT. However, the influence of iCBT on self-efficacy and physical activity was mostly mediated by improvements in depressive symptoms.

**Trial Registration:**

ClinicalTrials.gov NCT02778074; https://clinicaltrials.gov/ct2/show/NCT02778074

## Introduction

Depression is a serious health problem in patients with cardiovascular disease (CVD) (ie, atrial fibrillation/atrial flutter, ischemic heart disease, and heart failure). It is estimated that 20% to 40% of CVD patients experience depressive symptoms, leading to reduced health-related quality of life and increased risk of worse cardiovascular outcomes [1].

One possible mechanism behind these negative effects is that depressive symptoms are associated with biological changes such as overactivation of the sympathetic drive, chronic activation of the hypothalamic-pituitary-axis (HPA), and increased inflammation, all of which contribute to atherosclerosis, myocardial injury, and cell death [1,2], thus worsening CVD. Behavioral mechanisms are also important. In patients with different chronic conditions including CVD, depressive symptoms have been linked to poorer performance of self-care activities such as medication adherence and physical activity [3]. Furthermore, unhealthy behaviors such as sedentary lifestyles and smoking are more prevalent among CVD patients when compared to individuals without depressive symptoms [4]. Behavioral mechanisms, such as self-care, appear to play a role just as large as biological ones or an even larger role in cardiovascular outcomes [5].

Self-efficacy has an important role in the performance of adequate self-care behaviors [6]. In brief, self-efficacy stems from Bandura’s social learning theory that was later renamed to social cognitive theory and refers to the beliefs of people in their capacity to carry out specific behaviors [6,7]. Self-efficacy influences how people feel about, think about, and motivate themselves. For example, people with low self-efficacy have difficulties in tolerating obstacles and give up easily when trying to accomplish self-care behaviors [8,9]. In CVD, low self-efficacy has been associated with more hyperlipidemia [10], whereas improvements in self-efficacy are associated with improved physical activity and healthy food choices [11,12]. A study on patients with heart failure [13] showed that higher levels of depressive symptoms led to lower self-efficacy, which in turn fully mediated lower treatment adherence. This result was based on cross-sectional data and cannot be interpreted as causal. However, a study based on the same patient population using a 6-month follow-up longitudinal design reported that an increase in self-efficacy and a decrease in depressive symptoms were associated with improvements in medical adherence [14]. Except for this study, there is a knowledge gap in the associations among changes in depressive symptoms, self-efficacy, and self-care activities in CVD patients [13]. Knowledge about these associations is important, as there have been requests for behavioral interventions that could improve self-efficacy, reduce depressive symptoms, and improve performance of self-care activities in CVD patients [14].

In a previous randomized controlled trial (RCT), we have reported that 9 weeks of internet-based cognitive behavioral therapy (iCBT) led to significantly lower levels of depressive symptoms compared to an online discussion forum (ODF) at the 9-week follow-up (–2.34, 95% CI –3.58 to –1.10, *P*<.001, and Cohen *d=*0.62) [15]. Furthermore, in a secondary analysis of data from the RCT, we found that iCBT improved physical activity and that a decrease in depressive symptoms needed to precede an increase in physical activity [16], but the effects of iCBT on self-efficacy and the influences of changes in self-efficacy on changes in depressive symptoms and physical activity were not evaluated. In the RCT, data regarding self-efficacy were also collected and therefore, we now aim to (1) evaluate if iCBT can improve self-efficacy in CVD patients with depression and (2) explore the relationship among changes in self-efficacy, depressive symptoms, and physical activity, as well as the influence of iCBT on these relationships.

## Methods

### Design and Study Participants

This study received funding in January 2015 (trial registration NCT02778074) . Participant recruitment took place between January 2017 and February 2018, and the main findings were published in 2019 [15]**.** Thus, this study is a secondary analysis of the data that were collected in an RCT evaluating the effect of a 9-week iCBT program on depressive symptoms in patients with CVD. A total of 144 CVD patients with at least mild depressive symptoms (ie, Patient Health Questionnaire-9 score>5) were included and randomized to 9 weeks of the iCBT (n=72) or the ODF (n=72) [15]. The iCBT program consisted of 7 modules, namely goal setting, psychoeducation, problem-solving, behavioral activation, part 1 (2 weeks) and part 2 (2 weeks), and a summary module. The program also included weekly homework assignments with feedback provided by nurses. The ODF consisted of 9 discussion topics moderated by a nurse. The discussion took place in writing.

### Ethics Approval

The regional ethical review board of Linköping in Sweden approved the study (reference number 2016/72-31).

### Assessments

Data for this analysis were collected at baseline and at follow-up at the end of the 9-week intervention period. All data were collected through questionnaires on the study website [15].

#### Self-efficacy

Self-efficacy was measured using the Swedish version of the General Self-efficacy Scale (GSES) [17]. The GSES consists of 10 items that are rated on a 4-point Likert scale ranging from 1meaning not at all true to 4 meaning exactly true. A higher score reflects higher self-efficacy. The instrument has proved a reliable and valid measure of self-efficacy in general populations [18] and has also been used in cardiac populations [10]. The Cronbach *α* for the GSES in this study was .93.

#### Depressive Symptoms

The Montgomery Åsberg Depression Rating Scale (self-rating version) (MADRS-S) was used to measure depressive symptoms. This instrument consists of 9 items rated on a 7-point scale with a maximum score of 54. Scores 13-19, 20-34, and >35 indicate mild, moderate, and severe depression, respectively [19]. The Cronbach *α* in this study was .78.

#### Physical Activity

The frequency and length of physical activity were measured using 2 modified items from the Physical Activity Questionnaire [20]. Frequency was scored between 0 (none of the days) to 3 (often, 5-7 days). Length was scored from 0 (0 minutes/day) to 4 (more than 60 minutes/day). In our analysis, we created a physical activity factor by multiplying the 2 items and this factor has been used in a previous paper published by our group [16]. In that study, physical activity was measured once a week from baseline to the 9-week follow-up, and the mean interweek correlation for the physical activity factor scale was 0.8 [16], suggesting it to be a stable and reliable measure of physical activity. Self-reports and single response items are considered reliable and valid estimates of physical activity [21,22].

### Statistical Analysis

Descriptive statistics was used to describe the study population. Continuous variables were described as means and SDs, and categorical variables were described as numbers and percentages. All analyses were performed on original data, thus including participants with complete data at the 9-week follow-up (n=127). Analysis of covariance was used to evaluate if there was a significant difference in self-efficacy scores at the 9-week follow-up between the iCBT and ODF groups after adjustment for self-efficacy scores at baseline. The Cohen *d* was calculated for evaluating the effect of iCBT on self-efficacy. The associations among the changes in self-efficacy, depressive symptoms, and physical activity were explored by structural equation modeling (SEM). For these analyses, we calculated scores representing changes in self-efficacy, depressive symptoms, and physical activity between the baseline and 9-week follow-up measurements. To explore the influence of iCBT on these associations, we also added iCBT as an independent variable in the final SEM model. The associations/relationships obtained by SEM were described using their standardized coefficients (*β*). The chi-square value, the root mean square error of approximation (RMSEA), and comparative fit index (CFI) were used as goodness of fit indices of the SEM models. An insignificant chi-square would indicate a good model fit. An overall RMSEA below 0.06 indicates a good fit whereas a CFI ≥0.95 is considered a good fit, indicating that at least 95% of the covariation in the data is reproduced by the model. *P*<.05 indicates a significant value. Statistical analyses were performed with SPSS version 25.0 (IBM Corp) and the LISREL8 software (Scientific Software International) [23].

## Results

### Population

The mean age of the study population was 63 years. Among the 144 participants, 90 (62%) were males; almost 29 (20%) lived alone, and another 29 participants (20)% described their financial situation as problematic. Few participants were current smokers (ie, 4/144, 3%) or drank more than 10 units of alcohol per week (ie, 7/144, 5%). The median number of medications was 5 and approximately 48 participants had more than 1 comorbidity. Table 1 presents the characteristic of the study participants. The mean and SD values at baseline for self-efficacy, depressive symptoms, and physical activity as measured by the GSES, MADRS-S, and physical activity factor were 27.2 (6.3), 17.8 (6.7), and 4.8 (5.3) respectively. None of the variables presented in Table 1 differed significantly between those randomized to iCBT or the ODF.

**Table 1:** Description of the study population at baseline.

Characteristics				Total N=144	iCBT^a^ (n=72)	ODF^b^ (n=72)	*P* value		
Age in years, mean (SD)				63 (12)	61 (13)	64 (11)	.12		
**Gender, n (%)**									.39
	Male			89 (62)	47 (65)	42 (58)			
	Female			55 (38)	25 (35)	30 (42)			
Living alone, n (%)				28 (19)	13 (18)	15 (21)	.67		
**Education, n (%)**									.31
	Elementary			19 (13)	7 (10)	12 (17)			
	Upper secondary/high school			37 (26)	16 (22)	21 (29)			
	Postsecondary education/college/university< 2 years			35 (24)	21 (29)	14 (19)			
	University≥2 years			53 (37)	28 (39)	25 (35)			
**Lifestyle, n (%)**									
	**Smoking**							.75	
			Never	69 (48)	33 (46)	36 (50)			
			Ex-smoker	70 (49)	35 (51)	33 (46)			
			Smoker	5 (3)	2 (3)	3 (4)			
	**Alcohol**							.32	
			≤4 units/week	109 (76)	51 (71)	58 (81)			
			5-9 units/week	27 (19)	17 (24)	10 (14)			
			≥10 units/week	8 (5)	4 (5)	4 (5)			
**Cardiovascular diagnosis, n (%)**									
	Myocardial infarction/angina			49 (34)	34 (47)	29 (40)	.4		
	Atrial fibrillation			65 (45)	40 (56)	41 (57)	.86		
	Heart failure			30 (21)	18 (25)	20 (28)	.70		
	>1 diagnosis			40 (28)	20 (28)	20 (28)	.73		
**New York Heart Association Class, n (%)**									
	I			41 (28)	23 (32)	18 (25)			
	II			53 (37)	25 (35)	28 (39)			
	III			50 (35)	26 (33)	26 (36)			
**Medications**									
	Antidepressants, n (%)			20 (14)	7 (10)	13 (18)	.15		
	Antiplatelets/anticoagulants, n (%)			128 (88)	63 (88)	65 (90)	>.99		
	Beta-blockers, n (%)			110 (76)	55 (76)	55 (76)	>.99		
	Diuretics, n (%)			33 (23)	14 (19)	19 (26)	.32		
	Mineral receptor antagonists, n (%)			15 (8)	5 (7)	6 (8)	.75		
	Nitroglycerine, n (%)			30 (21)	15 (21)	15 (21)	>.99		
	RAAS^c^ blockade, n (%)			69 (48)	34 (47)	35 (49)	.86		
	Statins, n (%)			69 (48)	36 (50)	33 (46)	.62		
	Number of medications, median (IQR)			5 (4-6)	5 (4-6)	5 (4-6)	.72		
**Comorbidities, n (%)**									
	Hypertension			76 (53)	36 (50)	40 (56)	.5		
	Diabetes			21 (15)	8 (11)	13 (18)	.24		
	Pulmonary disease			15 (10)	7 (10)	8 (11)	.78		
	Transischemic attack/stroke			19 (13)	9 (12)	10 (14)	.81		
	Cancer			16 (11)	7 (10)	9 (12)	.6		
	>1 comorbidity			39 (27)	17 (24)	22 (31)	.35		
**Depressive symptoms, mean (SD)**									
	MADRS-S^d^			17.8 (6.7)	18 (7.2)	17.7 (6.2)	.31		
**Self-efficacy, mean (SD)**									
	General Self-efficacy Scale			27.2 (6.3)	27.0 (6.3)	27.4 (6.3)	.9		
**Physical activity, mean (SD)**									
	Physical activity factor			4.8 (3.5)	5.0 (3.7)	4.7 (3.4)	.7		

^a^iCBT: internet-based cognitive behavioral therapy.

^b^ODF: online discussion forum.

^c^RAAS: renin-angiotensin-aldosterone system.

^d^MADRS-S: Montgomery Åsberg Depression Rating Scale (self-rating version)

### Effect of the iCBT on Self-efficacy

At the 9-week follow-up, the mean self-efficacy scores and SD values for the iCBT group and the ODF group were 29.9 (5.8) and 28.2 (6.4), respectively. After adjustment for baseline scores, analysis of covariance showed a significant difference in the increase of self-efficacy in favor of iCBT (1.67, *P*=.04). The Cohen *d* was 0.27, indicating a small significant effect of iCBT on self-efficacy.

### Associations Among Self-efficacy, Depressive Symptoms, and Physical Activity

**Figure 1:**
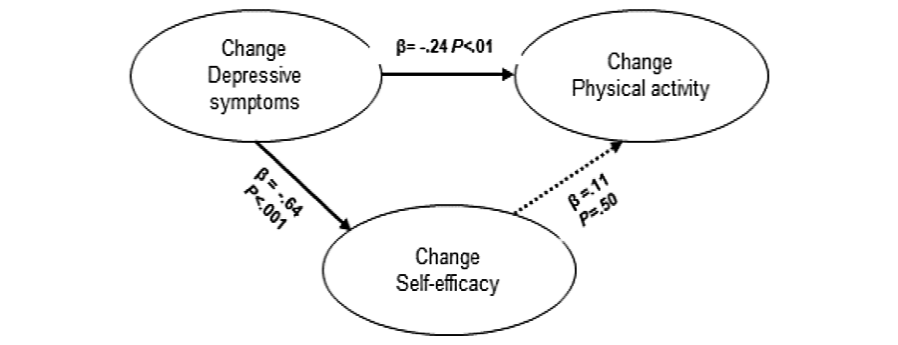
Model of the associations between changes in depressive symptoms, physical activity and self-efficacy based on the model by Maeda et al [13]. The associations in the model are described with the standardized coefficients (*β*). *P*<.05 indicates a significant value. The dotted line indicates a nonsignificant association. The fit of the model is perfect (*χ*^2^_0_=0, *P*>.99, CFI=1, and RMSEA=0). CFI: comparative fit index; RMSEA: root mean square error of approximation.

For analyzing the associations among the changes in self-efficacy, depressive symptoms, and physical activity, we first explored an SEM model based on the mediation model reported by Maeda et al [13]. They initially analyzed the association between depression and medical adherence and then that between depression and self-efficacy, which were both statistically significant. After adding the association between self-efficacy and medical adherence to the model, the significant association between depressive symptoms and medical adherence disappeared. Thus, self-efficacy served as a full mediator between depression and medical adherence. Our model showing the relationships among depression, physical activity, and self-efficacy (Figure 1) had a perfect fit (*χ*^2^_0_=0, *P*>.99, CFI=1, and RMSEA=0), which indicates that the model completely explained the correlations. We found a significant direct effect (*β=*–.24, *P*<.01) among changes in depression, physical activity, and self-efficacy (*β=*–.64, *P*<.001). However, the association between the change in self-efficacy and change in physical activity was weak and not statistically significant (*β*=.11, *P*=.5). Consequently, in our model, self-efficacy was not a mediator between changes in depressive symptoms and physical activity.

**Figure 2:**
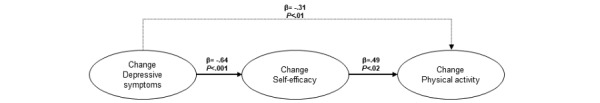
Simplex model describing the associations between changes in depressive symptoms, self-efficacy and physical activity. The model states that a change in depressive symptoms leads to a change in self-efficacy, which in turn leads to a change in physical activity. The thin grey dotted line indicates a significant indirect association between a change in depressive symptoms and a change in physical activity mediated by a change in self-efficacy. The associations in the model are described with the standardized coefficients (*β*). *P*<.05 indicates a significant value. The fit of the model is perfect (*χ*^2^_0_=0, *P*>.99, CFI=1, and RMSEA=0). CFI: comparative fit index; RMSEA: root mean square error of approximation.

However, the findings reported by Maeda et al [13] may also be interpreted as a simplex model, assuming that a change in depressive symptoms leads to a change in self-efficacy, which in turn leads to a change in physical activity. Given this assumption, self-efficacy could serve as a mediator between depressive symptoms and physical activity (Figure 2). This model had a perfect fit (*χ*^2^_0_=0, *P*>.99, CFI=1, and RMSEA=0). A summary of the associations is shown in Table 2. The model shows that a change in depressive symptoms has a significant relationship with the change in self-efficacy (*β=*–.64, *P*<.001), and a change in self-efficacy is significantly related to a change in physical activity (*β*=.49, *P*<.02). There was also an indirect association between the change in depressive symptoms and change in physical activity in which the change in self-efficacy served as a mediator (*β=*–.31, *P*<.01).

**Figure 3:**
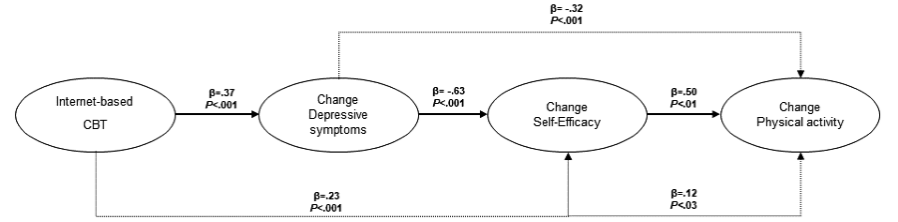
Simplex model describing the associations among changes in depressive symptoms, self-efficacy, and physical activity as a function of iCBT. The dotted lines indicate significant and indirect associations between iCBT and changes in self-efficacy and physical activity as well as between the changes in depressive symptom and physical activity. The associations in the model are described with the standardized coefficients (*β*). *P*<.05 indicates a significant value. The fit of the model is good (*χ*^2^2=1.49, *P*=.47, CFI=1, and RMSEA=0). CFI: comparative fit index; iCBT: internet-based cognitive behavioral therapy; RMSEA: root mean square error of approximation.

Furthermore, adding iCBT to our simplex model (Figure 3) showed that as expected, the iCBT had a direct and significant association with the changes in depressive symptoms (*β*=.37, *P*<.001) (*χ*^2^_2_=1.49, *P*=.47, CFI=1, and RMSEA=0 ). Table 3 summarizes these associations. The iCBT was also significantly and indirectly associated with the changes in self-efficacy (*β*=.23, *P*<.001) and physical activity (*β*=.12, *P*=.03). We also explored a reversed model in which the iCBT had a direct effect on the change in self-efficacy, which in turn influenced the changes in depressive symptoms and physical activity. The fit of the model was poor (*χ*^2^_2_=10.35, *P*=.005, CFI=0.82, and RMSEA=0.196), and the modification indices of the LISREL analysis strongly supported the model presented in Figure 3.

Performance accomplishment has been discussed as an important precursor of self-efficacy; if a person is successful at tasks, the feeling of efficacy will increase [6,7], which may indicate that an increase in self-efficacy leads to an increase in physical activity, which in turn increases self-efficacy. We added such a recursive association to the model presented in Figure 3. The fit of the model was good (*χ*^2^_2_=1.49, *P*=.47, CFI=1, and RMSEA=0) and showed that the relationship between self-efficacy and physical activity was significant (*β*=.5, *P*<.001) whereas the relationship between physical activity and self-efficacy was not (*β*=.27, *P*=.11). Accordingly, a significant recursive association was not found.

**Table 2:** Summary of the simplex model exploring the associations among changes in depressive symptoms, self-efficacy, and physical activity using the standardized coefficients (*β*).

		Change in depressive symptoms	Change in self-efficacy
**Change in self-efficacy**			
	*β*	–.64	—^a^
	*P* value	<.001	—
**Change in physical activity**			
	*β*	–.31	.49
	*P* value	<.001	.01

^a^Not applicable.

**Table 3:** Summary of the simplex model exploring the association of internet-based cognitive behavioral therapy with the changes in depressive symptoms, self-efficacy, and physical activity using the standardized coefficients (*β*).

		Internet cognitive behavior therapy	Change in depressive symptoms	Change in self-efficacy
**Change in depressive symptoms**				
	*β*	.37	—^a^	—
	*P* value	<.001	—	—
**Change in self-efficacy**				
	*β*	–.23	–.63	—
	*P* value	<.001	.001	—
**Change in physical activity**				
	*β*	–.12	–.32	.5
	*P* value	<.03	<.001	.01

^a^Not applicable.

## Discussion

### Principal Findings

The main findings of this study were that iCBT improved self-efficacy. However, the influence of iCBT on the improvement in self-efficacy and physical activity was mediated by improvements in depressive symptoms. Thus, self-efficacy was a mediator between improvements in depressive symptoms and physical activity.

In this study, we investigated the influence of self-efficacy on the changes in depressive symptoms and self-care through physical activity (ie, an aspect of autonomous self-care) [24]. To our knowledge, there is a lack of such studies involving CVD patients. In one of the few studies, Shen et al [14] showed that changes over 6 months in depressive symptoms (*β=*–.15, *P*=.05) and self-efficacy (*β*=.34, *P*<.001) were associated with medical adherence in patients with heart failure. However, in that study, only approximately 44% of the participants showed increased levels of depressive symptoms, and this may underestimate the results reported. Another study involving patients with heart failure reported that depression was negatively indirectly associated with poorer self-care through poorer self-efficacy [25], but this was a cross-sectionally designed study with no possibility to detect changes. Our study was performed on CVD patients with at least mild depression, and we found that a change from the baseline depressive symptoms was directly associated with the change in self-efficacy; this was indirectly associated with changes in physical activity, and thus self-efficacy had a mediating role in the relationship between changes in depressive symptoms and changes in physical activity. This highlights the importance of improvements in depressive symptoms in CVD patients. Such improvements are likely to be followed by improvements in self-efficacy and self-care, such as physical activity.

However, for such improvements to take place, an intervention is most likely needed. There have been requests for behavioral interventions that promote improvement of depressive symptoms, self-efficacy, and self-care in CVD patients [14]. In previous studies, we have found that an iCBT program for depression in CVD patients can improve depressive symptoms and increase physical activity [15,16]. This study now adds that iCBT can improve self-efficacy in depressed CVD patients as well. However, as observed in Figure 3, the impact of iCBT on self-efficacy is indirectly mediated by improvements in depressive symptoms. In Figure 3, the total effect of self-efficacy on physical activity is *β*=.5, but most of this is explained by the change in depressive symptoms (*β=*–.32); thus, the unique effect of self-efficacy on physical activity is *β*=.18 This suggests that only a minor part of the mediating role of self-efficacy between depressive symptoms and physical activity is related to self-efficacy itself. The model in which we analyzed if iCBT first led to changes in self-efficacy and then to changes in depressive symptoms was not valid, suggesting that improvements in depressive symptoms precede the increase in self-efficacy and physical activity. Moreover, we also explored if a change in self-efficacy leads to a change in physical activity, which in turn leads to changes in self-efficacy (ie, performance accomplishment). However, changes in physical activity had no significant effect on changes in self-efficacy (*β*=.27, *P*=.11).

Most digital interventional studies in cardiac rehabilitation focus on physical activity and counseling, with less focus on the core components such as psychological management [26]. However, our study suggests that in CVD patients with depressive symptoms, a digital intervention designed to improve self-efficacy and self-care such as physical activity should primarily focus on psychological management of depressive symptoms, for example, using iCBT. This does not contradict that the fact that such an intervention also includes physical activity counseling in the management of CVD and depression, and this can motivate the patients to perform physical activity when being involved in the iCBT program.

A limitation of this study is that this was a secondary analysis of data, and it did not primarily intend to investigate the effect of iCBT on self-efficacy or to explore the associations among changes in depressive symptoms, self-efficacy, and physical activity. Furthermore, data regarding physical activity were collected using only self-reports and not through objective measurements. This may have underestimated the level of physical activity measured [27] and may explain the nonsignificant association between self-efficacy and physical activity in Figure 1. However, in CVD patients with depression, there is a knowledge gap in the relationships among the changes in depressive symptoms, self-efficacy, and self-care such as physical activity and the influence of iCBT on these relationships. Therefore, we believe that despite the limitations, the results of this study provide interesting and useful information.

### Conclusions

The iCBT program was more effective than the ODF in increasing self-efficacy in CVD patients. An increase in self-efficacy was the mediator between improvements in depressive symptoms and physical activity. Improvements in depressive symptoms mediated most of the influence of iCBT on the improvements in self-efficacy and physical activity. The findings are preliminary and replication in larger samples is needed.

## Abbreviations

CVD: cardiovascular disease
CFI: comparative fit index
GSES: General Self-efficacy Scale
HPA: hypothalamic-pituitary-axis
iCBT: internet-based cognitive behavioral therapy
MADRS-S: Montgomery Åsberg Depression Rating Scale
ODF: online discussion forum
RCT: randomized controlled trial
RMSEA: root mean square error of approximation
SEM: structural equation modeling
